# The measurement and therapeutic implications of circulating tumour cells in breast cancer

**DOI:** 10.1038/sj.bjc.6602871

**Published:** 2005-11-29

**Authors:** J B Smerage, D F Hayes

**Affiliations:** 1 Department of Internal Medicine, Breast Oncology Program, University of Michigan Comprehensive Cancer Center, Ann Arbor, MI 48109, USA

**Keywords:** breast cancer, circulating tumour cells, prognosis, tumour marker, therapeutic monitoring

## Abstract

Circulating tumours cells (CTCs) represent an important biologic link in the spread of breast cancer from primary to metastatic disease. CTCs are strong predictors of prognosis in patients with metastatic breast cancer. Research to date has focused on development of methods with adequate sensitivity and specificity to reproducibly identify these rare events. Future research will focus on the biologic phenotypes of these cells with goals to understand mechanisms of metastasis, to identify novel therapeutic targets, and to monitor response to therapy.

The detection of circulating tumour cells (CTCs) in peripheral blood has been of interest for over a century (Ashworth, 1869). The existence of these cells fits nicely with the model of haematogenous spread in the development of metastatic disease and has the promise of providing a better understanding of the biology of metastasis. Current assays available to the practicing breast oncologist are largely based upon the presence or absence of these cells, but in the future it is hoped that biologic characteristics of CTCs, such as protein or mRNA expression, will be used in risk assessment, tailoring of treatment, monitoring of response, and development of novel therapeutic agents for patients with breast cancer. For the purpose of this review, we will limit our discussion to identification of circulating epithelial tumour cells in peripheral blood from patients with breast cancer.

## METHODS OF DETECTION

Much of the research over the past decade has focused on development of methods with sufficient sensitivity and specificity to detect CTCs, which are rare events occurring at a frequency of approximately one tumour cell per 1 × 10^5−7^ peripheral blood mononuclear cells (Ross *et al*, 1993). Methods to identify CTCs must distinguish between epithelial and haematopoietic cells in blood. Secondarily, it may be desirable, although not necessarily essential, to distinguish between cancer and normal epithelial cells. Selection based upon physical properties such as morphology, size, and weight have limitations in both sensitivity and specificity. In the 1950–1960s, identification relied upon morphology using light microscopy (Engell, 1959), but many of the identified cells are believed to be false positives due to artifacts of preparation and by the misclassification of leukocyte precursors (Christopherson, 1965). Cell separation by weight, as accomplished by the use of ficoll gradient, is associated with a significant loss in CTCs, with a recovery of only 10–65% cultured tumour cells spiked into whole blood (Choesmel *et al*, 2004; Rolle *et al*, 2005). In comparison, immunomagnetic separation techniques have approximately 85% recovery (Witzig *et al*, 2002; Allard *et al*, 2004).

With the advent of antibody and nucleic acid technologies, investigators turned to biologic properties such as protein expression and mRNA expression to identify CTCs in whole blood. Immunohistochemistry, immunofluorescent microscopy, and flow cytometry techniques have allowed significant progress in CTC research, but each of these in isolation continues to have both biological and technical limitations. Many of the antibodies directed at epithelial and breast cancer eptitopes are known to also stain haematopoietic cells, including EpCAM (Choesmel *et al*, 2004), cytokeratins (Racila *et al*, 1998), MUC-1 (Brugger *et al*, 1999), and TAG-12 (Ahr *et al*, 1999). Some of these false positives are concentration-dependent, and can be minimised by reducing the antibody concentration (Ahr *et al*, 1999). Nonspecific immunohistochemical staining of plasma cells can also occur due to nonspecific alkaline phosphatase reactions against the kappa and lambda light chains on the cell surface (Borgen *et al*, 1998). False positive rates range from 22 to 61% and vary based upon the antibody and the staining methodology. Several changes including optimisation of antibody concentration, selection of more specific antibodies and the use of directly labeled fluorescent monoclonal antibodies have improved some of these issues.

CTCs have also been indirectly identified using methods such as RT-PCR and PCR. RT-PCR has been used to detect breast cancer or epithelial associated mRNA transcripts such as cytokeratins, EGFR, mammoglobin, MUC-1, beta-HCG, c-Met, GalNac-T, MAGE-3, and others (Ring *et al*, 2004). However, as with immunological strategies, RT-PCR has also been hampered by false positives results in samples from normal volunteers and from patients with haematologic malignancies (Ring *et al*, 2004). These false positives stem from multiple sources, including issues with laboratory technique, primer selection, and illegitimate expression of the target genes in leukocytes. Interestingly, cytokeratin 19 and CEA expression can be induced in leukocytes by cytokines and growth factors (Jung *et al*, 1998; Goeminne *et al*, 1999). RT-PCR has also had problems with variable sensitivity. To increase sensitivity, some investigators are now testing strategies in which CTCs are identified by the presence of at least one mRNA transcript out of a panel of two or more tumour-associated transcripts (Taback *et al*, 2001; Ring *et al*, 2005). PCR has been used to detect free DNA within plasma. However, PCR has also had difficulty with poor specificity. This is due in part to the longer half-life of DNA in plasma when compared to mRNA. As a result it is unclear whether the free DNA that is amplified from plasma is from CTCs in the circulation or if the DNA is being shed from primary tumours, metastatic tumours, or from normal tissue (Ring *et al*, 2004). Concerns over assay specificity with PCR may be overcome by the identification of tumour specific DNA modifications, such as with methylation-specific PCR primers (Fiegl *et al*, 2005). However, this does not resolve the question of the origin of the amplified DNA. RT-PCR and PCR continue to have methodologic hurdles to overcome, but they hold promise in the effort to increase the sensitivity and specificity of CTC detection.

Published reports for the variety of CTC detction systems focus primarily on sensitivity, specificity, and correlation with stage. These reports vary dramatically in methodology, including the methods and targets used for CTC isolation and visualisation, definition of positive samples, and cohort size. They also vary in the amount and type of data presented, and as a result the ability to directly compare studies is limited. Specificity in many of these studies is low due to selection of a low threshold of positivity (one or more CTC). The most recent reports have used higher thresholds, which result in improved specificity with only mild losses in sensitivity (Cristofanilli *et al*, 2004; Ring *et al*, 2005).

The major finding of these studies is that the number of positive patients and the absolute numbers of CTCs per patient rise as clinical stage rises. The sensitivity in early stage disease continues to be low, resulting in CTC numbers that are not significantly different from those seen in control patients (Almokadem *et al*, 2005). This observation has limited the clinical use of CTCs to the metastatic setting in which CTCs can be reproducibly found in at least 50% of patients with metastatic breast cancer (Racila *et al*, 1998). Witzig *et al* (2002) purified CTC using immunomagnetic isolation against EpCAM and immunofluorescence against cytokeratin in 14–20 ml of blood. They identified ⩾2 CTCs in 64% of patient samples and ⩾5 CTCs in 52% of patient samples. In all, 76% of metastatic breast cancer patients had a least one detectable CTC and 8% of patients with node positive disease had at least one detectable CTC. None of the patients with node negative breast cancer had CTCs. None of the normal control samples had CTCs. Cristofanilli *et al* (2004) isolated CTCs using immunomagnetic isolation against EpCAM and immunofluorescence against cytokeratin in 7.5 ml of blood from patients with metastatic disease. They identified ⩾2 CTCs in 61% of pretreatment patient samples, and ⩾5 CTCs in 49% of pretreatment samples. None of the normal control samples had >2 CTCs, 1% had 2 CTCs, and 7% had 1 CTC.

Progress is now being made through the use of combined methods. An example is the CellSearch™ assay (Immunicon Corp., Hungtinton Valley, PA, USA), which is the only CTC assay to receive FDA clearance. This system combines biologic isolation techniques with biologic detection techniques, and uses an automated system to decrease intersample variability. CTCs are partially purified by immunomagnetic separation based upon expression of either EpCAM. Other investigators have used immunomagnetic separation based upon cytokeratin expression (Hu *et al*, 2003). The CellSearch system then visualises the CTCs by immunofluorescent microscopy. Other investigators have utilised immunohistochemistry, flow cytometry or RT-PCR as the method of detection and quantification after immunomagnetic isolation (de Cremoux *et al*, 2000; Ring *et al*, 2004). Many systems, including CellSearch, now routinely exclude non-specifically stained leukocytes via use of antibody stains directed against leukocyte-specific antigens such as CD45. Reproducibility has also been increased through the used of automated sample preparation and automated microscopy. The CellSearch system has been shown to be highly accurate and reproducible (Allard *et al*, 2004). Blood samples spiked with standardised numbers of cultured human breast cancer cells demonstrate a linear recovery over a range of 5–1142 cells (correlation coefficient *R*^2^=0.99), with an average recovery of >85% at each level. There was also strong agreement between duplicate samples (correlation coefficient *R*^2^=0.975) and between independent operators reviewing the same digital images (correlation coefficient *R*^2^=0.994).

## CLINICAL UTILITY OF CTCS

There are many potential clinical applications for CTCs in breast cancer including screening, predicting which patients with early stage disease will recur despite adjuvant therapy, monitoring for recurrence after adjuvant therapy, estimating prognosis in metastatic disease, predicting which drug is most likely to be efficacious for metastatic disease, and monitoring therapy for metastatic disease. However, good data only exist for establishing prognosis in metastatic breast cancer, and there is preliminary data for monitoring therapy in metastatic breast cancer. Only two studies have correlated the presence of immunopurified CTCs with clinical outcome.

Gaforio *et al* (2003) evaluated a heterogeneous population of patients spanning the clinical contexts of neoadjuvant therapy, adjuvant therapy, and metastatic disease. Utilising Kaplan/Meier PFS and OS curves, they found that patients with elevated CTCs prior to therapy had worse PFS (*P*=0.058) and OS (*P*=0.003). However, they did not stratify for disease stage, making interpretation of the data difficult. In addition, at the time of publication neither of the medians for PFS or OS had been reached.

**Figure 1:**
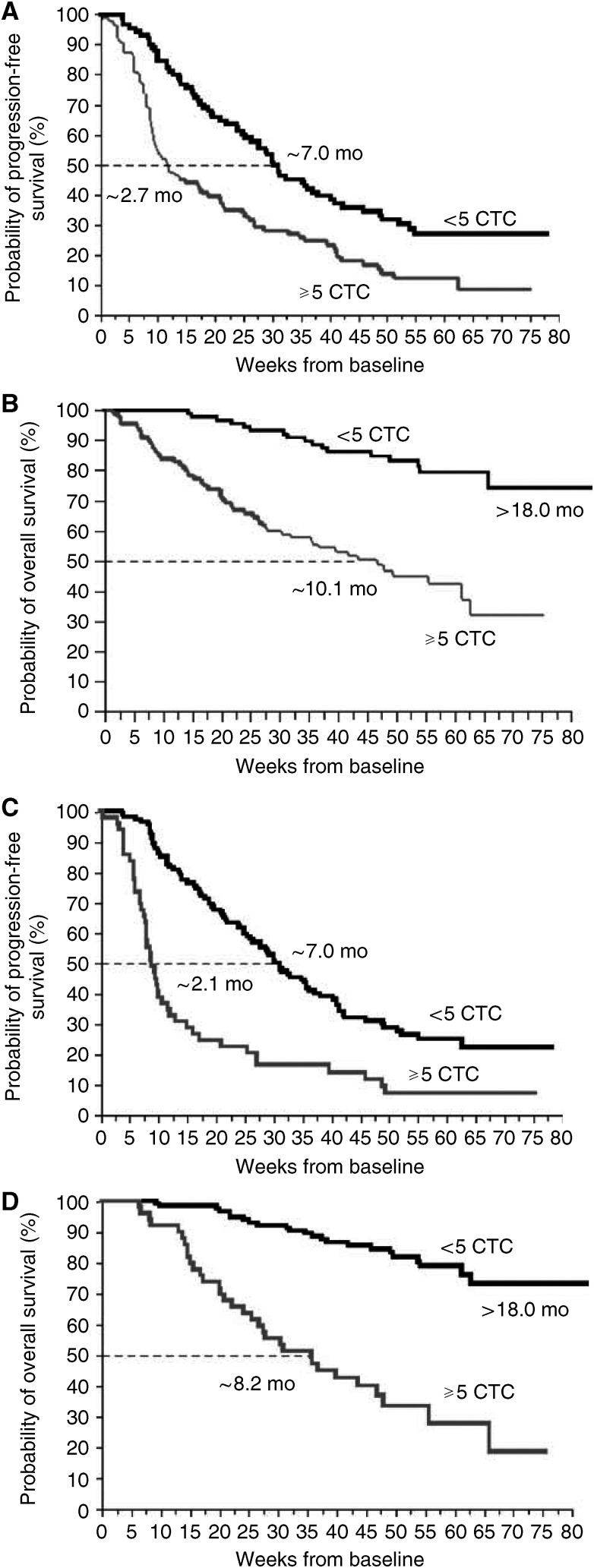
Kaplan–Meier curves demonstrating differences in PFS (**A** and **C**) and OS (**B** and **D**) based upon high and low risk CTC classifications. Patients with elevated CTCs at baseline (**A** and **B**) and at first follow-up after one cycle of therapy (**C** and **D**) have significantly worse median PFS and OS compared to the corresponding patients with low CTCs. Adapted with permission from (Cristofanilli *et al*, 2004).

Similarly, but in a much more rigorous fashion, Cristofanilli *et al* (2004, 2005) demonstrated that CTCs are highly prognostic in the metastatic setting . In a prospective, double-blind, multi-centre trial, 177 patients with metastatic breast cancer who were beginning a new therapy were evaluated. The trial utilised independent training and validation sets. Based upon the training set, elevated CTCs were defined as ⩾5 CTC per 7.5 ml of whole blood. Elevated CTCs at baseline predicted extremely short median PFS and OS of 3 months and 10 months, respectively. This is in comparison to patients with low/negative CTCs in whom PFS and OS were 7 months and 22 months, respectively (Figure 1). Thus baseline CTCs identify a group of high-risk patients. Even more interesting, CTC values obtained after one cycle of therapy predicted which patients were likely on ineffective therapy. Patients with elevated CTCs after one cycle of therapy had median PFS and OS of approximately 2.1 months and 8.2 months, respectively when measured from baseline (Figure 1). In contrast, median PFS and OS of 7.0 months and 22 months, respectively, were observed in the group with low CTCs. These differences were highly statistically significant (*P*<0.001) for patients receiving chemotherapy but not for patients receiving hormonal therapies.

Approximately 50% of patients with metastatic breast cancer do not have easily measurable disease. Therefore, the same investigators conducted a continuation study of 46 patients with bone-only disease, and CTCs were found to have similar prognostic significance (Budd *et al*, 2005). Patients with elevated baseline CTCs experienced a median PFS of 4.4 months and OS of 19 months compared to 9.5 (*P*=0.44) and >20 (*P*=0.25) months, respectively, in patients with low CTCs. At first follow-up median PFS were 3.5 and 14.4 months in the high- and low-risk groups, respectively (*P*=0.032). The medians for the OS endpoints have not yet been reached.

Although not a focus of this review, it is worth noting that the related phenomenon of micrometastatic disease in bone marrow has also been demonstrated to be a strong prognostic factor when evaluated at the time of definitive surgical therapy in early stage breast cancer (Braun *et al*, 2000, 2005). The number of epithelial cells in the bone marrow appears to be higher than in the peripheral blood, increasing the sensitivity of the assay in early stage breast cancer. As a result, such an assay may be useful in the risk assessment and treatment decision-making in the adjuvant setting.

## CURRENT CLINICAL STATUS OF CTCS

The recent study by Cristofanilli and colleagues strongly suggests CTCs could aid in the evaluation and monitoring of patients with metastatic breast cancer. The CellSearch assay has been cleared by the FDA and is commercially available for establishing prognosis in patients with metastatic disease. Current data appear to apply to patients with metastatic breast cancer initiating chemotherapy but not hormonal therapy. In selected patients, the prognostic value of CTCs may be helpful in determining the aggressiveness of therapy. CTC enumeration may be most helpful in selected patients with nonmeasurable disease since the onset of progression is particularly hard to determine in these patients. For patients in whom the radiographic and clinical information is inconclusive, the prognostic value of CTCs may allow a clinician to make a more informed decision about whether currently therapy is working and whether to switch therapies.

## FUTURE DIRECTIONS

In follow-up of the Cristofanilli data, a prospective randomised clinical trial has been developed in the Southwest Oncology Group (SWOG S0500) to test whether women with metastatic breast cancer and elevated CTCs at first follow-up after starting first line chemotherapy benefit from switching early to an alternate therapy compared to waiting for signs of clinical progression.

**Figure 2:**
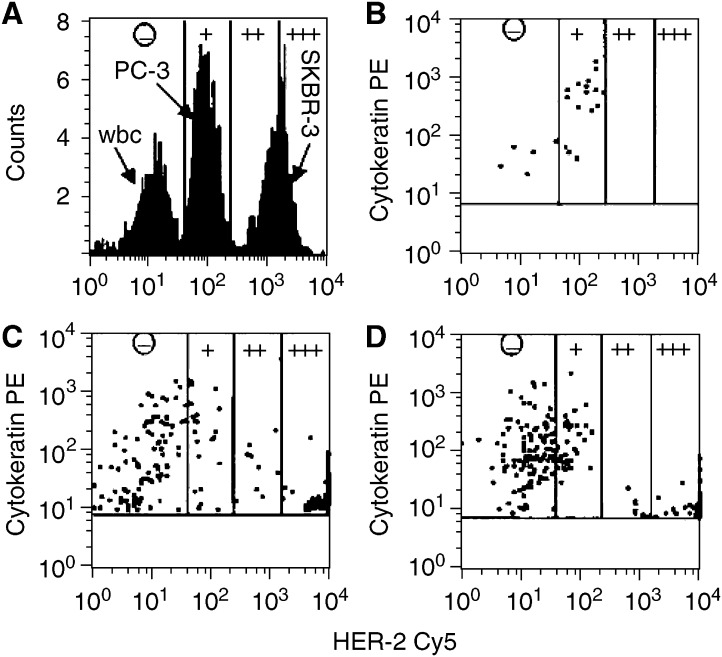
Quantification of HER-2 density on cell lines and on CECs of three breast cancer patients by flow cytometry. (**A**) HER-2 expression of leukocytes, PC3 cells and SKBR-3 cells immunomagnetically selected from 5 ml of blood and gated on size, CD45 expression, and cytokeratin expression. The expression levels of HER-2 were subdivided into four categories (−, +, ++, +++), based on the quantitative assessment of HER-2 expression on PC3 and SKBR-3 cells. (−) designates no expression or less than 5000 receptors (WBC); (+) designates expression between 5000 and 50 000 receptors (PC-3); (++) designates expression between 50 000 and 500 000 receptors; and (+++) designates expression of more than 500 000 receptors (SKBR-3). (**B**–**D**) show the expression of cytokeratin and HER-2 on CECs from three patients with breast cancer. Only the CECs are shown in the panels. Reproduced with permission from (Hayes *et al*, 2002).

In addition to enumeration, an exciting area of CTC research involves the phenotyping and expression profiling of CTCs. In this regard, one might consider evaluation of CTCs as a real-time biopsy. For most patients with metastatic breast cancer, the disease is internal, making it technically difficult and often risky and morbid to perform one biopsy, much less serial biopsies. It is possible that serial evaluation of CTCs, requiring only a simple blood test, may permit monitoring of drug targets during treatment. Furthermore, a better understanding CTC biology is likely to reveal previously unavailable information about the mechanisms of metastasis. For example, genetic changes can be detected in CTCs, including abnormal telomerase activity (Soria *et al*, 1999), allelic loss and/or amplification of multiple oncogenes not seen in normal control populations (Austrup *et al*, 2000), and aneuploid changes in cellular chromosome content based upon FISH analysis similar to those seen in the primary tumour (Fehm *et al*, 2002). Additionally, cancer-associated protein expression by CTCs can be detected, such as HER2 (Figure 2) (Hayes *et al*, 2002). Interestingly this report also suggests an inverse relationship between the level of HER2 expression and the expression of cytokeratin. Investigators have also demonstrated early successes in gene expression profiling (Smirnov *et al*, 2005) and multiplex RT-PCR (O’Hara *et al*, 2004) from CTCs. As each of these methodologies becomes more sophisticated, our ability to isolate, detect, and phenotype these cells will continue to improve.

If CTCs are placed in the broader concept of micrometastatic or minimal residual disease, it is interesting to note that not all patients with isolated tumour cells in regional lymph nodes, peripheral blood, or bone marrow demonstrate recurrence of their breast cancer. There are many possible explanations for this observation. Given the methodological limitations, many of these cells may simply be false positive events. However, the absence of elevated CTCs in normal populations and the presence of cytological abnormalities suggest otherwise. Thus some CTCs may be clinically significant while others may be biologically irrelevant. In other words, CTCs may differ in their proliferative and metastatic potential. This is consistent with the growing hypothesis of tumour stem cells (Al-Hajj *et al*, 2003; Dontu *et al*, 2004). If this hypothesis is true, then many micrometastatic cells, including CTCs may not be capable of continual self-renewal. This suggests that current therapies are targeting the ‘differentiated’ cells rather than the cancer stem cell. Work is ongoing to try to identify circulating tumour stem cells.

## CONCLUSIONS

CTCs undoubtedly play an important role in the development of distant metastases in breast cancer as well as other solid tumours. As a result of the rarity of these cells, our understanding of their biology remains in its infancy. However, many new techniques for the isolation and detection have been developed over the past decade, and CTCs are now known to be a strong prognostic factor in metastatic breast cancer. Investigators continue to develop more sophisticated methods to phenotype CTCs. This should lead to the discovery of new therapeutic targets as well as the ability to monitor the modulation of these targets during clinical trials and eventually as a part of standard of care therapy.
